# Congenital Superior Lumbar Hernia (Grynfeltt-Lesshaft Hernia) Associated With Orofacial, Limb, and Rib Anomalies: A Neonatal Case Report

**DOI:** 10.7759/cureus.110354

**Published:** 2026-06-06

**Authors:** Ayed Askar, Shrouk F Mohamed, Ahmed Alsweed

**Affiliations:** 1 Surgery, Idlib University Hospital, Idlib, SYR; 2 Surgery, Alexandria University, Alexandria, EGY; 3 Pediatric Surgery, American Hospital Dubai, Dubai, ARE

**Keywords:** case report, congenital superior lumbar hernia, grynfeltt-lesshaft hernia, neonatal hernia, upper lumbar hernia

## Abstract

Congenital superior lumbar hernia (Grynfeltt-Lesshaft hernia) is an extremely rare defect of the posterior abdominal wall in neonates and is frequently associated with other congenital anomalies. We report a one-month-old female presenting with a right-sided lumbar swelling since birth. Clinical examination revealed a large, reducible mass measuring 8 × 10 cm in the superior lumbar triangle. Associated anomalies included a complete right-sided cleft lip, right thumb hypoplasia with radial deviation, and absence of the lower right thoracic ribs. Abdominal ultrasound demonstrated bowel loops within the hernial sac without signs of obstruction or strangulation. Further imaging confirmed rib agenesis without cardiac, renal, or vertebral abnormalities. The patient underwent successful open tension-free mesh hernioplasty. Postoperative recovery was uneventful, with good healing and no recurrence at one-month follow-up. This case highlights the importance of early diagnosis and treatment of lumbar hernia, particularly when accompanied by other congenital anomalies.

## Introduction

Congenital lumbar hernias (CLHs) are rare compared to acquired hernias, accounting for approximately 20% of all lumbar hernias that are typically acquired later in life. Among the few congenital cases, those involving the superior lumbar triangle (Grynfeltt-Lesshaft triangle) are particularly uncommon in neonates [1,2].

Most reported cases of congenital lumbar hernia are associated with other congenital deformities. More than 70% of these cases were syndromic and often occurred in conjunction with lumbocostovertebral syndrome (LCVS), which involves deformities with the spine, ribs, and the genitourinary system. The association between congenital lumbar hernias and other congenital disabilities highlights the importance of early detection, investigation of associated anomalies, and genetic testing [3]. Imaging, including ultrasound (US) and computed tomography (CT) scans, helps characterize the hernia [4].

Surgical repair is the definitive treatment. The timing and technique of surgery depend on defect size, tissue quality, and associated anomalies. Significant defects with poor muscular support may require prosthetic reinforcement to achieve a tension-free repair [2,3,5].

We report this case because of the exceptional rarity of congenital lumbar hernia of the superior lumbar triangle in a neonate, particularly in association with multiple anomalies, and to highlight the clinical decision-making surrounding early mesh-based repair in this age group. This plays a crucial role in enhancing the sparse literature available on the subject.

## Case presentation

A female neonate aged one month, born at term by standard vaginal delivery, with a birth weight of 2.2 kg and a current weight of 5 kg, presented with a congenital swelling in the right lumbar region noted since birth. The mass was soft, reducible, and enlarged during episodes of crying, consistent with the clinical presentation of a hernia. This case was documented in May 2025. 

**Figure 1:**
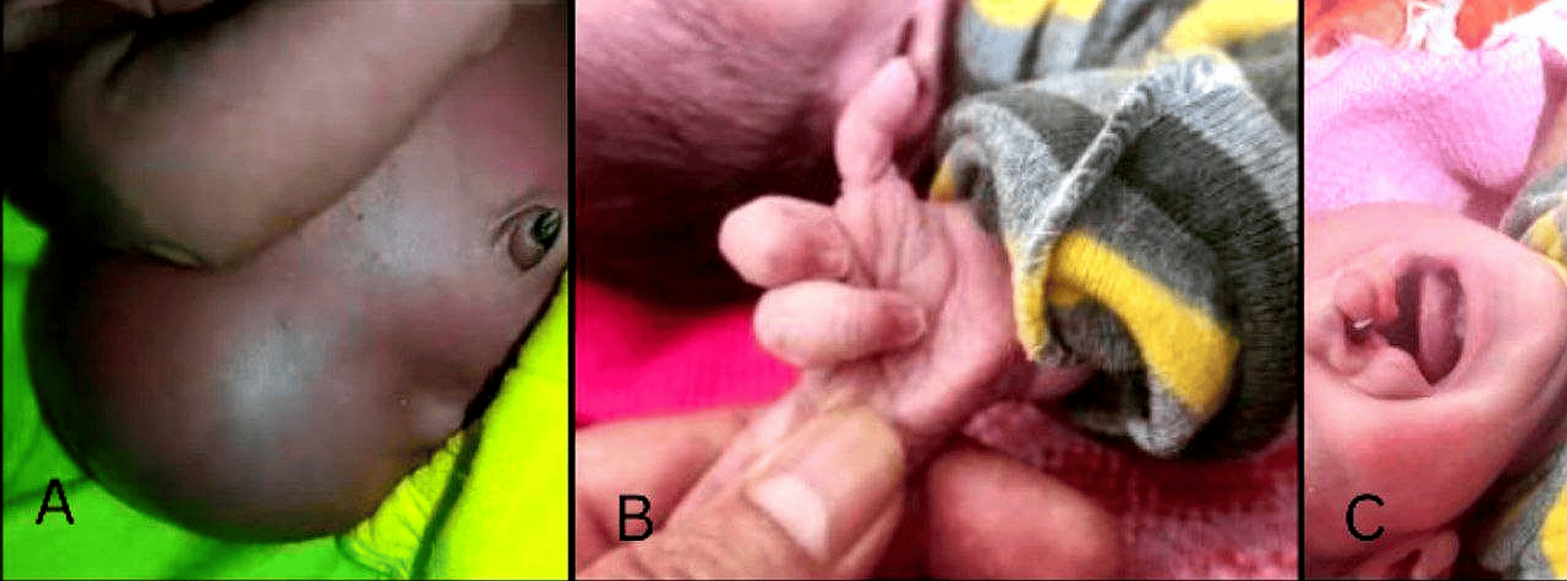
Clinical photographs showing characteristic features of a neonate presenting with a lumbar hernia and associated congenital anomalies (A) Large lumbar hernia appearing as a soft, reducible swelling in the lower back region; (B) Congenital thumb deformity; (C) Cleft lip

Physical examination indicated that the swelling was significant, measuring 8 × 10 cm, and was located in the superior lumbar triangle in the right lumbar region, which is known as Grynfeltt's triangle. She also had other congenital deformities, including a complete right-sided cleft lip and right thumb hypoplasia with radial deviation and limited abduction (Figure 1).

An abdominal ultrasound revealed bowel loops within the hernial sac, with no evidence of intestinal obstruction or strangulation. Screening for associated anomalies was performed using echocardiography, abdominal ultrasonography, and chest radiography. Echocardiography showed no structural cardiac abnormalities. Abdominal ultrasonography demonstrated normal renal anatomy with no associated anomalies. Chest radiography confirmed agenesis of the lower right thoracic ribs, with no vertebral deformities or thoracic organ abnormalities. Genetic consultation was advised for further syndromic evaluation.

Laboratory studies were performed to rule out additional underlying conditions. The complete blood count revealed a white blood cell count of 10,800/mm³ (reference range: 5,000-15,000/mm³), hemoglobin of 12.0 g/dL (reference range: 10-14 g/dL), and platelet count of 335,000/mm³ (reference range: 150,000-450,000/mm³). Serum electrolytes showed potassium of 5.4 mmol/L (reference range: 3.5-5.0 mmol/L) and calcium of 9.34 mg/dL (reference range: 8.5-10.5 mg/dL).

The patient was prepared to undergo general anesthesia for surgical correction. For right lumbotomy, a right superior lumbar incision was performed obliquely. The fascial defect measured approximately 8 × 10 cm intraoperatively. Surrounding musculature was markedly hypoplastic, and the absence of supporting ribs resulted in poor posterior thoracic wall stability.

**Figure 2:**
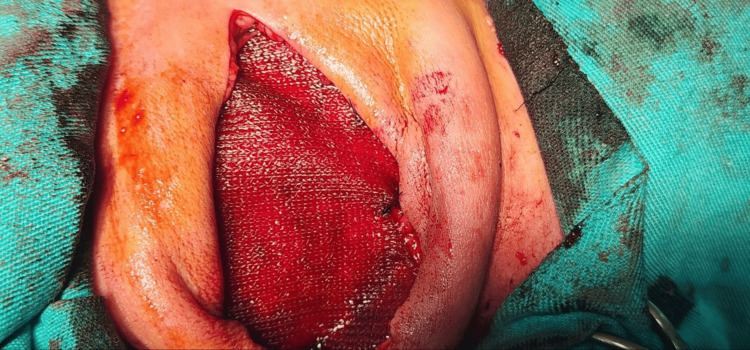
Intraoperative photograph illustrating the placement of the mesh over the hernia defect

The bowel was completely reduced to the abdominal cavity. Because primary closure would have resulted in excessive tension, a tension-free mesh hernioplasty using a polypropylene mesh was performed. The mesh was secured to the surrounding musculature in a supra-aponeurotic position. Layered closure was completed with adequate hemostasis (Figure 2).

On the first postoperative day, the patient resumed oral intake; she was discharged in good health the day following surgery. Follow-up evaluations at one week and one month showed appropriate wound healing with no evidence of infection or recurrence. Long-term follow-up has been planned to monitor thoracic growth and mesh behavior.

## Discussion

Congenital lumbar hernias are rare, especially when occurring through the superior lumbar triangle. As the majority of lumbar hernias are acquired in adults due to trauma, occurrences in newborns are somewhat unusual in pediatric surgery [2,5]. 

A notable characteristic of congenital lumbar hernias is their frequent association with other developmental anomalies. In fact, as many as two-thirds of documented incidents in the literature are related to musculoskeletal or urogenital anomalies. LCVS is a widely established condition characterized by absent or hypoplastic ribs, spinal deformities such as hemivertebrae or scoliosis, and aplasia of the posterior abdominal wall musculature. These musculoskeletal deficits often reveal the anatomical vulnerabilities that contribute to the development of hernias [6].

Additional associations include features overlapping with vertebral anomalies, anal atresia, cardiac defects, tracheoesophageal fistula, renal abnormalities, and limb deformities (VACTERL). Although rare, some reported cases of lumbar hernias include combinations of these defects [5,6].

Although elective repair of uncomplicated congenital lumbar hernias is often deferred until 6-12 months of age, early intervention was chosen in this patient due to several high-risk anatomical features. The defect was large, progressively enlarging, and contained bowel loops. Furthermore, agenesis of the lower right ribs produced thoracic wall instability and deficient muscular support, increasing the likelihood of progressive enlargement and future incarceration. These factors made delayed repair potentially unsafe.

Primary anatomical closure is preferred when feasible; however, in this case, the defect size and severe muscular hypoplasia rendered tension-free primary repair impossible. For this reason, prosthetic reinforcement was required. Also, the use of mesh in neonates remains controversial due to concerns about interference with musculoskeletal growth. However, in large defects with absent ribs and deficient musculature, mesh reinforcement may be the only viable option to achieve a durable repair [2]. Long-term outcomes with mesh reinforcement are generally favorable, with most patients maintaining durable repair as they grow. Nevertheless, careful follow-up is essential, as some children may require revision or adjustment of the repair to accommodate thoracic and abdominal wall growth, particularly in cases with extensive rib agenesis or deficient musculature [2,5].

Minimally invasive techniques have recently been reported as an alternative approach, particularly in older children. A few case reports have documented successful laparoscopic or laparoscopic-assisted repairs in infants. However, experience with this method remains limited, and concerns persist about the adequacy of closure, risk of organ injury, and long-term outcomes in growing children [2,7].

## Conclusions

This case study highlights the importance of quickly diagnosing and surgically treating congenital lumbar hernias. Preoperative evaluation is critical for detecting associated anomalies and guiding treatment strategies. Early intervention can help prevent complications like incarceration or progressive hypertrophy. Furthermore, continuous monitoring across diverse disciplines is crucial for assessing both growth and development, along with the long-term outcomes of surgical interventions.
